# The Royal Marsden Hospital Score Independently Predicts Overall Survival in Patients with De Novo Metastatic Renal Cell Carcinoma Treated with First-Line Tyrosine Kinase Inhibitors: A Multicenter Retrospective Analysis

**DOI:** 10.3390/jcm15103613

**Published:** 2026-05-08

**Authors:** Tolga Doğan, Semra Taş, Taliha Güçlü Kantar, İrfan Karahan, Atike Gökçen Demiray, Musa Barış Aykan, Burcu Yapar Taşköylü, Melek Özdemir, Arzu Yaren, Nuri Karadurmuş, Gamze Gököz Doğu

**Affiliations:** 1 Department of Medical Oncology, Denizli State Hospital, 20010 Denizli, Türkiye; talihaguclu@hotmail.com (T.G.K.); melekozdemir@hotmail.com (M.Ö.); 2 Department of Medical Oncology, Faculty of Medicine, Pamukkale University, 20070 Denizli, Türkiye; semratasdr@gmail.com (S.T.); gokcenakaslan@gmail.com (A.G.D.); drburcuyapar@gmail.com (B.Y.T.); arzu_yaren@yahoo.com (A.Y.); ggd2882@gmail.com (G.G.D.); 3 Department of Medical Oncology, Zonguldak Bülent Ecevit University, 67100 Zonguldak, Türkiye; irfan_karahan@yahoo.com; 4 Department of Medical Oncology, Gülhane Training and Research Hospital, University of Health Sciences, 06010 Ankara, Türkiye; musabarisaykan@gmail.com (M.B.A.); drnkaradurmus@yahoo.com (N.K.)

**Keywords:** metastatic renal cell carcinoma, RMH score, IMDC, tyrosine kinase inhibitors, overall survival, prognostic model

## Abstract

**Background:** Accurate prognostic stratification is essential for clinical decision-making in metastatic renal cell carcinoma (mRCC). Although the International Metastatic RCC Database Consortium (IMDC) model is widely used, its discriminatory capacity may be limited in specific clinical subpopulations. The Royal Marsden Hospital (RMH) score, based on serum albumin, lactate dehydrogenase, and the number of metastatic sites, has not been evaluated as a prognostic tool in patients with de novo mRCC receiving first-line tyrosine kinase inhibitor (TKI) therapy. **Methods:** We retrospectively analyzed the data of 149 patients with de novo metastatic renal cell carcinoma who received first-line TKI therapy (pazopanib, sunitinib, or cabozantinib) at two tertiary oncology centers in Turkey. Overall survival (OS) and progression-free survival (PFS) were estimated using the Kaplan–Meier method. Univariate and multivariate Cox proportional hazards regression analyses were performed to identify independent prognostic factors. **Results:** The median OS and PFS were 23.1 months (95% CI: 19.4–26.8) and 9.4 months (95% CI: 7.0–11.8), respectively. The OS showed a stepwise decline across RMH risk groups, ranging from 40.7 months in patients with an RMH score of 0 to 8.6 months in those with an RMH score of 3. In the multivariate analysis, the RMH score (HR 1.29, 95% CI: 1.03–1.61; *p* = 0.026) and sarcomatoid differentiation (HR 2.01, 95% CI: 1.09–3.72; *p* = 0.025) were independently associated with worse OS. The IMDC score did not retain independent prognostic significance (*p* = 0.129). The RMH score was not significantly associated with PFS after multivariable adjustment, and the IMDC score was not significantly associated with PFS in univariate analysis and was therefore not entered into the multivariable PFS model. **Conclusions:** The RMH score independently predicted overall survival in patients with de novo mRCC receiving first-line TKI therapy, whereas the IMDC score did not retain independent prognostic significance in this cohort. Given its simplicity and reliance on objective parameters, the RMH score may provide complementary prognostic information in this patient population; however, external validation in independent cohorts is required before broader clinical implementation can be considered.

## 1. Introduction

Renal cell carcinoma (RCC) represents the most frequently encountered malignant tumor of the kidney, which continues to constitute a substantial public health challenge globally; approximately 20–30% of patients present with synchronous metastatic disease at the time of diagnosis, and the outcomes remain persistently unfavorable despite meaningful progress in systemic treatment [1,2,3]. This degree of clinical variability underscores the critical need for robust prognostic classification to inform therapeutic decision-making and support individualized patient counseling.

Tyrosine kinase inhibitor (TKI)-based regimens served as the cornerstone of treatment in metastatic renal cell carcinoma (mRCC) for well over a decade [4]; however, immune checkpoint inhibitor (ICI)-based combination regimens have emerged as the preferred first-line approach for the majority of patients in clinical practice [2,5,6,7,8]. TKI monotherapy remains a viable option in certain clinical situations, especially for patients who cannot undergo immunotherapy or in healthcare systems where access to combination treatments is still restricted. A recent real-world study enrolling 1538 patients with mRCC demonstrated that TKI monotherapy constituted nearly 42% of all first-line treatment regimens administered in routine clinical practice [9]. In these scenarios, precise prognostic evaluation before treatment initiation is vital to support tailored clinical decision-making.

Among the various prognostic tools available, the International Metastatic Renal Cell Carcinoma Database Consortium (IMDC) score stands as the most commonly employed model in the clinical evaluation of patients with metastatic renal cell carcinoma [10]. The model integrates six clinical and laboratory parameters—Karnofsky performance status, time elapsed from diagnosis to treatment initiation, hemoglobin concentration, corrected serum calcium level, neutrophil count, and platelet count—and stratifies patients into favorable, intermediate, and poor prognostic categories. The IMDC model has been shown to be a dependable prognostic indicator across numerous independent cohorts and continues to retain its relevance in the era of therapies utilizing ICIs [11]. Nevertheless, its discriminatory capacity remains modest, with concordance index values typically ranging from 0.63 to 0.70 [10]. Moreover, certain factors within the model—especially the time between diagnosis and the start of treatment—may have limited ability to differentiate outcomes within cohorts consisting solely of patients with newly diagnosed metastatic disease, who generally begin systemic therapy shortly after diagnosis. These shortcomings indicate that complementary prognostic methods could prove valuable for certain patient subgroups.

The Royal Marsden Hospital (RMH) prognostic score was initially created to enhance patient selection for phase I oncology trials and has subsequently been assessed across various cancer types [12]. The evaluation is determined by three objective criteria: a serum albumin level below 3.5 g/dL, a serum lactate dehydrogenase (LDH) level exceeding the normal upper limit, and the existence of more than two metastatic sites. Each criterion contributes one point, resulting in a composite score ranging from 0 to 3, where higher scores suggest a worse prognosis. In contrast to the IMDC model, the RMH score draws exclusively on laboratory and imaging-derived data, omitting clinician-rated performance status, which may help to minimize interobserver variability. A comprehensive systematic review and meta-analysis involving more than 127,000 patients across a range of tumor types corroborated the association between higher RMH scores and diminished survival outcomes [13].

According to Hahn et al., the RMH score demonstrated better prognostic accuracy than the IMDC model in a cohort of patients participating in phase I trials for metastatic renal cell carcinoma [14]. However, phase I cohorts are highly selective and often include heavily pretreated patients, which limits the direct applicability of those findings to patients receiving standard first-line TKI therapy in routine practice. In addition, the IMDC model includes the interval between diagnosis and treatment initiation, a variable that may have limited discriminatory value in patients presenting with de novo metastatic disease. Whether the RMH score can provide useful prognostic information in this specific subgroup has not been systematically examined. Therefore, we conducted a multicenter retrospective study to evaluate the prognostic value of the RMH score in patients with de novo mRCC receiving first-line TKI therapy and to compare its performance with that of the IMDC model, with overall survival as the primary endpoint.

## 2. Materials and Methods

### 2.1. Study Design and Patient Population

This multicenter retrospective cohort study was conducted at two tertiary oncology centers in Turkey: the Pamukkale University Faculty of Medicine (Denizli) and the Gülhane Training and Research Hospital (Ankara). The medical records of patients diagnosed with metastatic renal cell carcinoma (mRCC) who received first-line tyrosine kinase inhibitor (TKI) therapy between January 2010 and December 2025 were retrospectively reviewed. Only patients presenting with metastatic disease at the time of the initial diagnosis (de novo metastatic disease) were included in this study.

### 2.2. Inclusion and Exclusion Criteria

Patients were included if they met the following criteria:

(1) Age ≥ 18 years;
(2) Histologically confirmed renal cell carcinoma of any subtype;
(3) Presence of metastatic disease at the time of initial diagnosis (de novo metastatic disease);
(4) Receipt of first-line TKI therapy (pazopanib, sunitinib, or cabozantinib);
(5) Availability of sufficient clinical and laboratory data required for the calculation of the RMH and IMDC prognostic scores.

Patients were excluded if they had received prior systemic therapy for metastatic disease, had incomplete clinical or laboratory data preventing prognostic score calculation, or had concurrent active malignancy. A total of 149 eligible patients met all inclusion criteria and had complete data available for analysis. A formal screening log was not prospectively maintained across both centers, which is a limitation of the retrospective design.

### 2.3. Prognostic Score Definitions

The International Metastatic RCC Database Consortium (IMDC) score was calculated according to the model described by Heng et al. [15]. The IMDC model incorporates six clinical and laboratory variables: Karnofsky performance status <80%, time from diagnosis to treatment < 1 year, hemoglobin level below the lower limit of normal, corrected serum calcium level above the upper limit of normal, neutrophil count above the upper limit of normal, and platelet count above the upper limit of normal. The Karnofsky performance status was not directly documented in the medical records of the participating centers; the ECOG performance status ≥ 2 was therefore used as the clinical equivalent of Karnofsky performance status < 80%, consistent with standard mapping in oncology practice. The frequency of each individual IMDC component is reported in Supplementary Table S1. Because all patients in this cohort presented with de novo metastatic disease, the IMDC component “time from diagnosis to treatment initiation < 1 year” was positive in all patients and therefore had no discriminatory value. Standard six-factor IMDC categorical stratification would consequently not yield a favorable-risk group in this cohort. For descriptive purposes only, we report the modified 5-factor IMDC descriptive categories derived from the remaining five components, excluding the universally positive time-to-treatment variable. These categories were not used as the primary analytic variable in any regression model.

The Royal Marsden Hospital prognostic score was calculated based on three parameters: serum albumin < 3.5 g/dL, serum lactate dehydrogenase (LDH) above the upper limit of normal, and the presence of >2 metastatic sites. Each parameter was assigned one point, resulting in a total score ranging from 0 to 3, with higher scores indicating worse prognosis. Pretreatment laboratory values obtained within 4 weeks before the initiation of first-line TKI therapy were used for the score calculation.

### 2.4. Outcome Measures

The primary endpoint of this study was overall survival (OS), characterized as the period from the commencement of first-line TKI therapy to death from any cause. The secondary endpoint, progression-free survival (PFS), was characterized as the interval from the commencement of treatment to either the initial confirmed disease progression according to RECIST version 1.1 criteria or death from any cause, whichever came first. Patients without an event were censored at the date of the last documented clinical contact [16].

### 2.5. Data Collection

Baseline clinicopathological data were obtained from electronic medical records and archived patient files. The recorded variables included age, sex, smoking status, ECOG performance status, histological subtype, presence of sarcomatoid differentiation, metastatic sites (lung, liver, bone, central nervous system, and soft tissue), number of metastatic regions, and first-line TKI agent.

Laboratory parameters, including complete blood count and serum biochemistry values (albumin, LDH, and calcium), were obtained within four weeks prior to the initiation of first-line TKI therapy and were used to calculate the RMH and IMDC prognostic scores.

### 2.6. Statistical Analysis

Continuous variables were represented as either the mean with the standard deviation (SD) or the median with the interquartile range (IQR), whereas categorical variables were described using frequencies and proportions. The Kaplan–Meier method was utilized to construct survival curves for OS and PFS, and group differences were evaluated using the log-rank test.

Univariate and multivariate Cox proportional hazards regression analyses were performed to identify independent prognostic factors associated with OS and PFS. The multivariable Cox proportional hazards model was constructed using a combination of a priori clinical considerations and statistical parsimony. Both the RMH and IMDC scores were included in the OS multivariable model by design as the primary study variables. For the PFS multivariable model, the same covariate selection approach was applied; the IMDC score did not meet the prespecified inclusion criterion in univariate PFS analysis (*p* = 0.743) and was therefore not entered into the multivariable PFS model. Additional covariates, including sex, sarcomatoid differentiation, prior nephrectomy, histological subtype, age, and performance status, were considered on the basis of the established clinical relevance in mRCC and retained only if compatible with a parsimonious final model given the available number of events. The results were reported as hazard ratios (HR) with corresponding 95% confidence intervals (CI). A two-sided *p*-value < 0.05 was considered statistically significant. All statistical analyses were performed using IBM SPSS Statistics for Windows, version 26.0 (IBM Corp., Armonk, NY, USA).

The follow-up duration was estimated using the reverse Kaplan–Meier method. The proportional hazards assumption was assessed for all variables included in the multivariable Cox model using Schoenfeld residuals, and no violation was detected (all *p* > 0.05). To formally assess the discriminatory performance of the RMH and IMDC scores, Harrell’s concordance index (C-index) and ROC curve analysis for overall survival were performed. Confidence intervals for the C-index were estimated using 2000 bootstrap resamples. Landmark AUC analysis was additionally performed at 12, 24, and 36 months to characterize the temporal pattern of model discrimination. The overall ROC curve analysis was performed using SPSS Statistics version 26.0, with binary vital status at last follow-up as the classification outcome. This non-time-dependent ROC approach does not formally account for censoring, and the resulting overall AUC values do not correspond to a specific time horizon. Landmark AUC analyses at 12, 24, and 36 months were performed using a binary landmark approach; patients who died before each landmark were classified as events, patients alive beyond the landmark were classified as non-events, and patients censored before the landmark were excluded from that specific analysis. Harrell’s C-index was used as the principal measure of survival discrimination for the censored time-to-event data and was calculated using the lifelines library in Python (version 3.11; Python Software Foundation, Wilmington, DE, USA) with 95% confidence intervals estimated from 2000 bootstrap resamples. To assess potential multicollinearity between the RMH and IMDC scores, variance inflation factors (VIFs) were calculated for all variables included in the multivariate model. Sensitivity analyses were additionally performed using separate Cox models, each including only one of the two prognostic scores. To account for treatment heterogeneity, the first-line TKI agent was included as a covariate in a sensitivity multivariable Cox model, with pazopanib as the reference category. An interaction term between the RMH score and TKI agent was also tested. The relatively broad enrollment period was chosen to obtain a cohort of patients with de novo metastatic disease treated with first-line TKI therapy; however, this design may have introduced temporal heterogeneity related to changes in clinical practice over time. Residual confounding and case-mix heterogeneity cannot be fully excluded in this observational study.

During the preparation of this manuscript, AI-assisted tools were used for language editing and writing assistance. The authors reviewed and edited all AI-assisted content and take full responsibility for the accuracy and integrity of the work.

### 2.7. Ethical Considerations

This study was approved by the Pamukkale University Non-Interventional Clinical Research Ethics Committee (decision date: 27 January 2026; decision number: E-60116787-020-823417) and conducted in accordance with the principles of the Declaration of Helsinki. Due to the retrospective design of the study and the use of anonymized data extracted from medical records, the requirement for informed consent was waived by the ethics committee.

## 3. Results

### 3.1. Patient Characteristics

A total of 149 patients with de novo metastatic renal cell carcinoma who received first-line tyrosine kinase inhibitor therapy were included in this analysis. Most tumors were of clear cell histology (73.2%), and sarcomatoid differentiation was present in 16 patients (10.7%). Using the modified five-factor IMDC descriptive categories that excluded the universally positive time-to-treatment component, 35.6% of patients were categorized as favorable, 54.4% as intermediate, and 10.1% as poor. These categories are reported for descriptive purposes only and do not represent the standard six-factor IMDC risk classifications. The distribution of RMH scores was 35.6% for score 0, 38.3% for score 1, 21.5% for score 2, and 4.6% for score 3. The baseline clinicopathological characteristics of the study population are summarized in Table 1.

### 3.2. Survival Outcomes

The median follow-up duration, estimated using the reverse Kaplan–Meier method, was 67.4 months (range: 0.5–106.0 months). Among the 50 patients alive at the time of analysis, the median follow-up was 20.9 months (range: 3.6–105.5 months). A total of 123 progression events and 99 deaths occurred over a maximum follow-up period of 106 months. Patients without an event were censored at the date of the last documented clinical contact, and no patients were lost to follow-up.

**Figure 1:**
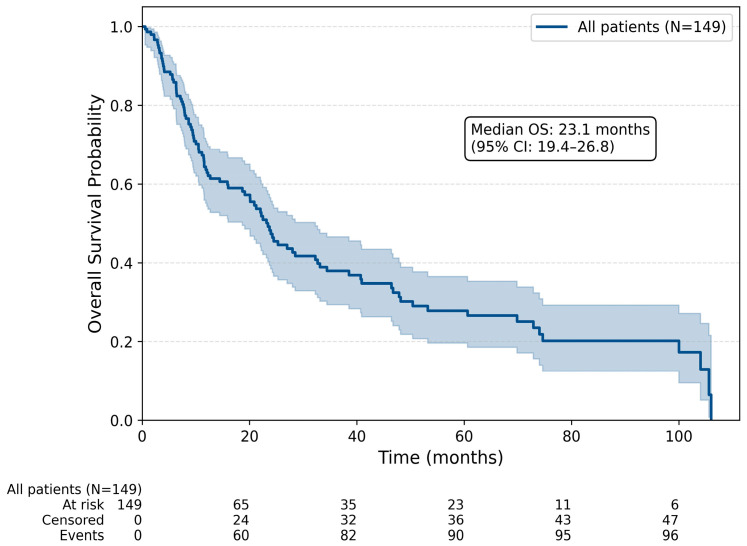
Kaplan–Meier curve for overall survival of the entire cohort (N = 149). Median OS: 23.1 months (95% CI: 19.4–26.8). The shaded area represents the 95% confidence interval.

The median progression-free survival (PFS) for the entire cohort was 9.4 months (95% confidence interval [CI], 7.0–11.8), and the median overall survival (OS) was 23.1 months (95% CI, 19.4–26.8; Figure 1).

**Figure 2:**
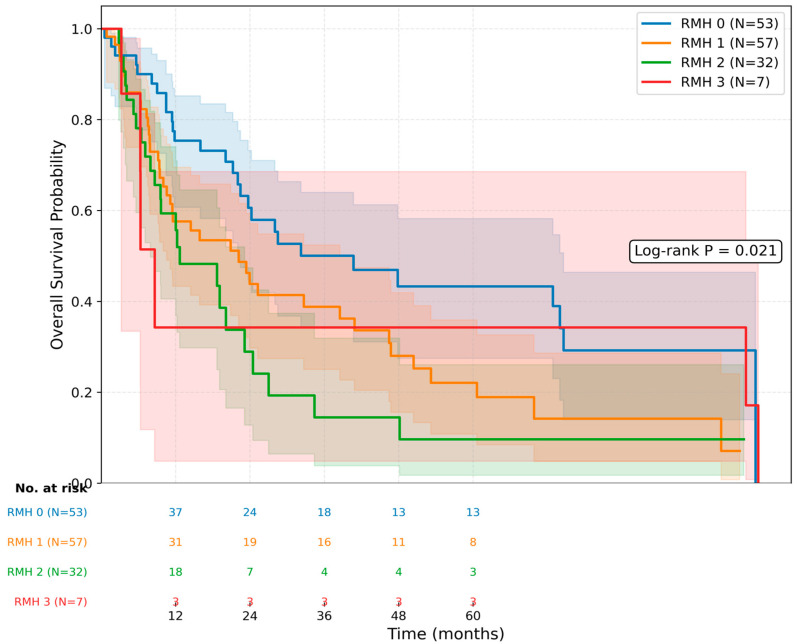
Kaplan–Meier curves for overall survival according to RMH score groups. Numbers at risk are shown below the figure. The shaded areas represent 95% confidence intervals. Log-rank *p* = 0.021.

**Figure 3:**
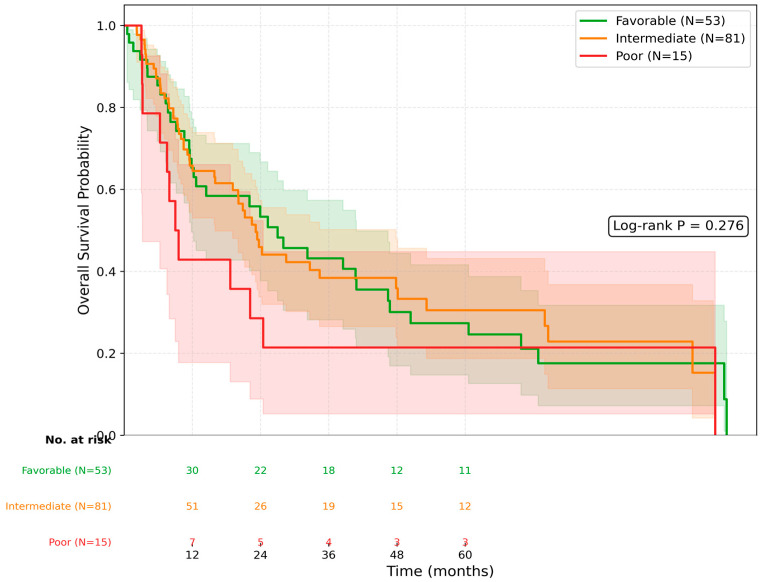
Kaplan–Meier curves for overall survival according to the modified five-factor IMDC descriptive categories, derived by excluding the universally positive IMDC component “time from diagnosis to treatment initiation < 1 year”. Numbers at risk are shown below the figure. The shaded areas represent 95% confidence intervals. Log-rank *p* = 0.276.

When stratified according to the Royal Marsden Hospital (RMH) prognostic score, a stepwise decrease in median overall survival was observed with increasing RMH scores. The median OS was 40.7 months (95% CI: 15.2–66.1) for patients with an RMH score of 0, 22.1 months (95% CI: 11.0–33.2) for a score of 1, 12.6 months (95% CI: 2.9–22.3) for a score of 2, and 8.6 months (95% CI: 6.0–11.2) for a score of 3 (Figure 2). Kaplan–Meier analysis according to the modified five-factor IMDC descriptive categories did not demonstrate significant separation in overall survival (log-rank *p* = 0.276; Figure 3).

### 3.3. Prognostic Factors for Overall Survival

Univariate and multivariate Cox proportional hazards regression analyses were performed to identify the factors associated with OS. In the univariate analysis, male sex (HR 1.39, 95% CI: 0.87–2.21; *p* = 0.165), non-clear cell histology (HR 1.30, 95% CI: 0.84–2.00; *p* = 0.242), and age (HR 1.01, 95% CI: 0.99–1.02; *p* = 0.431) were not significantly associated with OS. Sarcomatoid differentiation showed a trend toward worse survival (HR 1.69, 95% CI: 0.93–3.07; *p* = 0.078).

In the multivariate model, sarcomatoid differentiation (HR 2.01, 95% CI: 1.09–3.72; *p* = 0.025) and the RMH score (HR 1.29, 95% CI: 1.03–1.61; *p* = 0.026) were independently associated with overall survival. Male sex (HR 1.45, 95% CI: 0.88–2.38; *p* = 0.141), the IMDC score (HR 1.16, 95% CI: 0.95–1.42; *p* = 0.129), and prior nephrectomy (HR 0.67, 95% CI: 0.42–1.05; *p* = 0.077) did not demonstrate independent prognostic significance. Detailed results of the Cox regression analysis for overall survival are presented in Table 2.

To formally assess the model discrimination, Harrell’s C-index and ROC curve analysis for overall survival were performed. The C-index was 0.595 (95% CI: 0.534–0.652) for the RMH score and 0.543 (95% CI: 0.481–0.602) for the IMDC score. The exploratory non-time-dependent ROC curve analysis yielded an AUC of 0.606 (95% CI: 0.510–0.702; *p* = 0.036) for the RMH score and 0.477 (95% CI: 0.382–0.571; *p* = 0.644) for the IMDC score. In landmark AUC analysis, the RMH score showed AUC values of 0.614, 0.634, and 0.629 at 12, 24, and 36 months, respectively; the corresponding values for the IMDC score were 0.554, 0.630, and 0.628. Multicollinearity assessment showed a VIF of 2.38 for the RMH score and 3.65 for the IMDC score; all remaining covariates had VIF values below 3. The Pearson correlation coefficient between the two scores was 0.270. In sensitivity analyses using separate Cox models, the RMH score remained independently associated with overall survival when modeled without IMDC (HR 1.30, 95% CI: 1.04–1.62; *p* = 0.022), whereas the IMDC score did not reach statistical significance when modeled without RMH (HR 1.21, 95% CI: 0.998–1.456; *p* = 0.053). In a sensitivity analysis adjusting for first-line TKI agent, the RMH score remained independently associated with overall survival (HR 1.27, 95% CI: 1.002–1.599; *p* = 0.048). The interaction between the RMH score and TKI type was not statistically significant (*p* = 0.382 for RMH × sunitinib; *p* = 0.560 for RMH × cabozantinib), indicating no evidence that the prognostic effect of the RMH score differed by treatment subgroup. In the same model, sunitinib was associated with shorter overall survival than pazopanib (HR 1.91, 95% CI: 1.21–3.01; *p* = 0.005). In a sensitivity analysis restricted to patients with clear cell histology (N = 108, 66 OS events; one patient with clear cell histology was excluded due to incomplete covariate data), the RMH score showed a directionally consistent association with overall survival (HR 1.17, 95% CI: 0.88–1.56; *p* = 0.283). Although statistical significance was not retained in this subgroup, the direction and magnitude of the effect estimate remained similar to those observed in the full cohort.

### 3.4. Prognostic Factors for Progression-Free Survival

**Figure 4:**
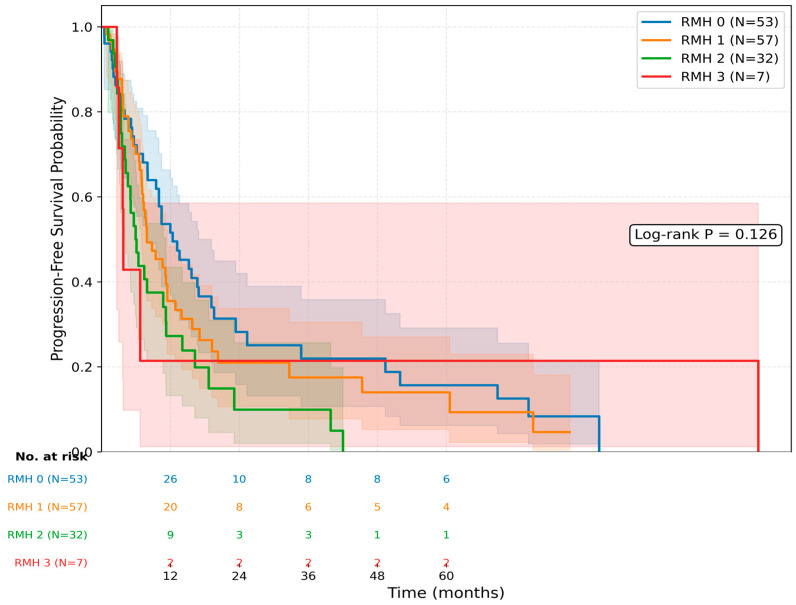
Kaplan–Meier curves for progression-free survival according to RMH score groups. Numbers at risk are shown below the figure. Shaded areas represent 95% confidence intervals. Log-rank *p* = 0.126.

Univariate and multivariate Cox proportional hazards regression analyses were also performed to evaluate factors associated with PFS. Kaplan–Meier analysis according to RMH score groups did not demonstrate significant separation in progression-free survival (log-rank *p* = 0.126; Figure 4). In the univariate analysis, male sex was associated with shorter PFS (HR 1.54, 95% CI: 1.02–2.34; *p* = 0.040). Sarcomatoid differentiation showed a non-significant trend toward worse PFS (HR 1.60, 95% CI: 0.92–2.78; *p* = 0.090), whereas age and non-clear cell histology were not significantly associated with PFS.

In the multivariate analysis, male sex (hazard ratio [HR], 1.53; 95% confidence interval [CI], 0.99–2.38; *p* = 0.057) and sarcomatoid differentiation (HR, 1.65; 95% CI, 0.95–2.87; *p* = 0.074) showed borderline associations with progression-free survival. The RMH score did not demonstrate a statistically significant association with progression-free survival (HR, 1.23; 95% CI, 0.99–1.52; *p* = 0.056). The IMDC score was not entered into the multivariable PFS model, as its univariate association with PFS did not meet the prespecified inclusion criterion (*p* = 0.743). The detailed results of the Cox regression analyses for progression-free survival are shown in Table 3. Notably, the multivariable association between the RMH score and PFS approached statistical significance (*p* = 0.056), in contrast to the clearer association observed for overall survival.

## 4. Discussion

In this multicenter retrospective study of patients with de novo metastatic renal cell carcinoma receiving first-line tyrosine kinase inhibitors, the Royal Marsden Hospital score emerged as an independent predictor of overall survival. In contrast, the widely used International Metastatic Renal Cell Carcinoma Database Consortium score did not retain prognostic significance in the multivariate model. These findings suggest that the RMH score may provide clinically meaningful prognostic information in this patient population and may complement existing risk stratification models. The median overall survival of 23.1 months and median progression-free survival of 9.4 months observed in our cohort are broadly consistent with outcomes reported in real-world cohorts of patients with mRCC treated with first-line TKI therapy, although they are somewhat lower than those reported in pivotal randomized trials that typically included more selective patient populations [4,17].

As outlined in the Introduction, the RMH score incorporates three objective parameters—serum albumin, lactate dehydrogenase, and the number of metastatic sites—that together capture the disease burden and systemic physiological reserve. Consistent with prior evidence from large meta-analytic data [13], higher RMH scores in our cohort were associated with a clear stepwise decline in overall survival, ranging from a median of 40.7 months in patients with a score of 0 to 8.6 months in those with a score of 3.

Previous studies evaluating the RMH score in metastatic renal cell carcinoma are limited. In a cohort of patients with mRCC enrolled in phase I clinical trials, Hahn et al. reported that the RMH score performed favorably relative to the IMDC model for both overall and progression-free survival [14]. This distinction is clinically relevant, as phase I cohorts are highly selective and often include heavily pretreated patients, whereas individuals receiving first-line TKI therapy in routine practice represent a broader population in whom baseline prognostic stratification may directly inform treatment planning. Our findings are consistent with these observations. In our cohort, the RMH score remained independently associated with overall survival in a multivariate analysis, whereas the IMDC score did not retain statistical significance. One potential explanation for this finding may relate to the composition of our cohort, which consisted exclusively of patients presenting with de novo metastatic disease. In this clinical setting, certain variables included in the IMDC model—particularly the interval between diagnosis and treatment initiation—may have reduced the discriminatory value, as systemic therapy is typically initiated shortly after diagnosis. By contrast, the RMH score relies solely on objective laboratory and disease burden-related parameters, which may provide more stable prognostic information in this patient population.

The heterogeneity of first-line TKI agents used in this cohort—pazopanib in 49.7%, sunitinib in 35.6%, and cabozantinib in 14.8% of patients—represents a potential source of treatment-related variability. In a TKI-adjusted sensitivity analysis, the RMH score retained independent prognostic significance, and formal interaction testing showed no evidence that its prognostic effect differed by TKI type. In the same model, sunitinib was associated with a shorter overall survival than pazopanib; however, in this retrospective cohort, this finding may reflect patient selection and treatment-era effects rather than a treatment-specific difference. This should be kept in mind when interpreting the prognostic findings across treatment subgroups.

The present findings should not be interpreted as establishing categorical superiority of the RMH score over the IMDC model. Rather, in this cohort, the RMH score showed independent prognostic significance and numerically more favorable, although modest, discrimination metrics for overall survival, while the IMDC score showed weaker and less consistent discrimination. These results support further evaluation of the RMH score in patients with de novo mRCC receiving TKI therapy, but they do not justify the replacement of established prognostic models without external validation in independent cohorts. The finding that the IMDC score did not retain independent prognostic significance in the multivariable model should be interpreted with caution and does not imply that the IMDC model is inferior as a prognostic tool. The IMDC model has been extensively validated across large and heterogeneous mRCC cohorts and remains the standard risk stratification framework in clinical practice. In our cohort, the attenuated performance of the IMDC score may partly reflect the composition of the study population. Because all patients in this cohort presented with metastatic disease at diagnosis, the interval between diagnosis and treatment initiation—one of the six IMDC components—likely had reduced discriminatory value in this setting. In addition, the moderate sample size and the inclusion of a correlated prognostic score in the same multivariable model may have further limited the ability to detect an independent contribution of IMDC. These considerations underscore the need for caution when interpreting comparative model performance in a single cohort.

The absolute discriminatory performance of both models was modest in this cohort. The C-index values overlapped substantially, and the landmark AUC estimates at 24 and 36 months were numerically close between the two models. These observations caution against overinterpretation of the comparative discrimination findings, and the RMH score should be regarded as a potential complement to, rather than a replacement for, existing risk stratification approaches, pending external validation.

The biological rationale underlying the prognostic value of the RMH score in metastatic renal cell carcinoma is supported by the established roles of its individual components. Serum LDH serves as an indicator of tumor metabolic activity and the overall extent of the disease, and it is strongly associated with the aberrant metabolic reprogramming of malignant cells underlying the Warburg effect. A systematic review and meta-analysis of 30 studies involving 6754 patients with metastatic RCC revealed that higher pretreatment LDH levels were linked to notably poorer overall survival (HR 2.15, 95% CI: 1.85–2.51) and progression-free survival (HR 1.76, 95% CI: 1.49–2.10), establishing LDH as a strong prognostic indicator for this condition [18].

The second element of the RMH score, serum albumin, acts as a comprehensive indicator of both nutritional health and the systemic inflammatory burden. Inflammation linked to cancer enhances the production of hepatic acute-phase proteins while reducing albumin synthesis, and low albumin levels have been repeatedly linked to inferior survival outcomes in patients with mRCC undergoing TKI treatment [19]. The third component, the number of metastatic sites, captures the anatomical scope of the disease spread. A comprehensive study involving 2027 mRCC patients undergoing targeted therapy from the IMDC database revealed that bone and liver metastases were linked to poorer overall survival [20], which persisted even after accounting for IMDC risk factors, with adjusted hazard ratios of 1.40 and 1.42, respectively. In our cohort, 65.1% of patients had the involvement of two or more metastatic sites, with bone metastases in 45.0% and liver metastases in 18.8%. Together, these three components capture complementary aspects of tumor biology—metabolic activity, systemic inflammatory–nutritional status, and metastatic burden—thereby explaining the prognostic relevance of the RMH score in advanced RCC.

In addition to the RMH score, sarcomatoid differentiation emerged as an independent predictor of worse overall survival in our cohort (HR 2.01, 95% CI: 1.09–3.72; *p* = 0.025), which aligns with earlier research characterizing sarcomatoid dedifferentiation as an aggressive tumor phenotype linked to resistance against VEGFR-targeted treatments and diminished survival in patients with metastatic RCC receiving first-line TKI therapy [21]. The effect size observed in our study was comparable to those in previous reports, further supporting the adverse prognostic impact of sarcomatoid differentiation in this setting.

Sarcomatoid differentiation is unlikely to be fully captured by a simple binary classification. A higher sarcomatoid component has generally been associated with more aggressive disease, particularly in patients treated with VEGFR-targeted therapy. At the same time, patients with sarcomatoid features may show different outcomes under contemporary ICI-based regimens, suggesting that the clinical meaning of sarcomatoid differentiation depends not only on its presence but also on its extent and the treatment context. In our cohort, only the presence or absence of sarcomatoid differentiation could be retrieved from the pathology reports, which should be kept in mind when interpreting the observed association between sarcomatoid differentiation and overall survival.

Male sex was associated with shorter progression-free survival in the univariate analysis (HR 1.54, 95% CI: 1.02–2.34; *p* = 0.040); however, this relationship did not persist after multivariate adjustment (*p* = 0.057). The borderline association between male sex and shorter PFS should be interpreted cautiously. Sex was not independently associated with survival after multivariable adjustment in our cohort, and this finding may reflect residual clinical heterogeneity rather than a direct prognostic effect. In our study, the type of histology classified as non-clear cell did not have a notable impact on survival rates. This finding aligns with previous research indicating that the histological subtype alone may not hold significant independent prognostic value when well-established clinical and laboratory risk factors are taken into account [10,14].

The difference between the OS and PFS findings deserves comment. While the RMH score was independently associated with overall survival, it did not demonstrate a statistically significant association with progression-free survival after multivariable adjustment, although the association approached statistical significance (*p* = 0.056). The IMDC score was not significantly associated with PFS in univariate analysis and was therefore not entered into the multivariable PFS model. One possible explanation is that PFS is more sensitive to treatment-specific and assessment-related factors, including the timing of radiologic evaluation and treatment-related decision-making. OS, in contrast, reflects the broader disease course and may be more closely linked to the baseline disease burden and host condition. Baseline markers of systemic inflammation, nutritional status, and tumor burden—which together underlie the RMH score—may be more closely aligned with long-term mortality than with the short-term tempo of radiologic progression. In this setting, the present findings suggest that the primary clinical utility of the RMH score may lie in overall survival stratification rather than progression prediction.

Several potential confounders merit explicit consideration. The ECOG performance status showed a trend toward worse overall survival in the univariate analysis and was considered during model building, though it was not retained in the final multivariable model; its potential residual influence should be acknowledged when interpreting the results. Prior nephrectomy showed a borderline association with better overall survival in the multivariable model (HR 0.67, 95% CI: 0.42–1.05; *p* = 0.077); its inclusion in the adjusted model means that the prognostic estimates for both scores were adjusted for this variable. Metastatic burden was incorporated directly into the RMH score through the number-of-metastatic-sites component, and its prognostic contribution is therefore represented within the score itself. These considerations should be kept in mind when interpreting the prognostic estimates for the RMH and IMDC scores.

Based on the present findings, the RMH score is not proposed as a replacement for the IMDC model, which remains the standard risk stratification tool in mRCC and is supported by extensive validation data. Rather, the RMH score may serve as a complementary prognostic tool in specific clinical contexts—particularly in patients with de novo metastatic disease receiving first-line TKI therapy, in whom the discriminatory value of certain IMDC components may be limited. The simplicity of the RMH score, based on three routinely available objective parameters, and its independent prognostic performance in the present cohort support its further evaluation alongside established risk stratification approaches, pending external validation in independent cohorts.

Several limitations of this study should be acknowledged. First, the retrospective design may be susceptible to selection bias and unmeasured confounding, as the treatment decisions and follow-up strategies were not fully standardized across the two participating institutions. In addition, the enrollment period spanned 15 years (2010–2025), during which the treatment landscape for metastatic RCC evolved substantially. Although this approach allowed us to assemble a clinically relevant TKI-treated cohort, a calendar effect related to changes in supportive care, imaging, and subsequent therapies cannot be excluded. Furthermore, because the study population was restricted to patients receiving first-line TKI monotherapy, the findings should not be directly extrapolated to patients treated with contemporary ICI-based combination regimens. Second, although the cohort size of 149 patients is comparable to that of previously reported single-center studies evaluating prognostic models in metastatic RCC, the sample size may have limited the statistical power for certain subgroup analyses, particularly for variables with relatively low prevalence, such as sarcomatoid differentiation (n = 16). Third, the extent of sarcomatoid differentiation was not quantified as a percentage of the tumor volume in pathology reports, which may have limited the ability to explore potential dose–response relationships between the sarcomatoid component and survival outcomes. Fourth, the heterogeneity of first-line TKI agents used in the cohort—pazopanib (49.7%), sunitinib (35.6%), and cabozantinib (14.8%)—may represent a source of treatment-related variability; although TKI-adjusted analyses and formal interaction testing supported the consistency of the RMH prognostic effect across treatment subgroups, the treatment assignment was not randomized and may have introduced residual confounding related to patient selection and treatment era. Fifth, detailed comorbidity data were not systematically available in a format suitable for standardized scoring, which precluded an adjustment for the baseline comorbidity burden. Individual IMDC risk factors were not entered separately into the multivariable model because the diagnosis-to-treatment interval had limited variation in this exclusively de novo cohort, and the treatment dose intensity was not recorded consistently across both centers. Sixth, our analysis was confined to two tertiary oncology centers within a single country, and the prognostic performance of the RMH score has not been externally validated—a major limitation requiring validation in independent and geographically diverse cohorts before broader clinical application can be considered. Seventh, detailed information on second- and later-line systemic therapies was not available in a sufficiently standardized format, precluding formal analysis of subsequent treatment sequences; accordingly, the potential influence of post-progression treatments, including later-line immune checkpoint inhibitor-based therapy, on overall survival cannot be excluded. Eighth, the cohort included 40 patients (26.8%) with non-clear cell histology, which may have introduced additional biological and treatment-related heterogeneity; in a sensitivity analysis restricted to clear cell RCC, the RMH score showed a directionally consistent association with overall survival, but statistical significance was not retained, and histology-specific validation studies are therefore warranted. In addition, formal calibration analysis was not performed, and no internal validation procedure beyond bootstrap estimation of the C-index confidence intervals was applied; given the size and retrospective design of the cohort, these additional model performance assessments were not considered sufficiently stable for robust interpretation. The total number of patients screened prior to applying the inclusion and exclusion criteria was not prospectively recorded across both centers, which limits the transparency of the patient selection process; because excluded patients could not be characterized systematically, the possibility of selection bias related to incomplete data cannot be fully excluded. These limitations should be considered when interpreting our findings, and prospective validation in larger multicenter cohorts is warranted. Formally time-dependent ROC analysis was not performed; therefore, the non-time-dependent overall AUC values based on vital status at last follow-up should be interpreted as exploratory and not as substitutes for survival-model discrimination metrics.

In conclusion, the findings of this multicenter retrospective study indicate that the Royal Marsden Hospital score independently predicts overall survival in patients with de novo metastatic renal cell carcinoma receiving first-line tyrosine kinase inhibitor therapy, whereas the IMDC score did not retain prognostic significance in this population. Sarcomatoid differentiation also emerged as an independent adverse prognostic factor, consistent with its established role as a marker of aggressive tumor biology. Based on three routinely available objective parameters—serum albumin, lactate dehydrogenase, and the number of metastatic sites—the RMH score represents a simple and reproducible complement to existing risk stratification approaches in this setting. Whether the RMH score retains prognostic value in patients receiving contemporary ICI-based combination regimens remains to be determined. External validation in independent and more diverse cohorts is required before broader clinical implementation can be considered, and prospective studies are needed to clarify its role in the evolving therapeutic landscape of metastatic renal cell carcinoma.

## Figures and Tables

**Table 1:** Baseline clinicopathological characteristics of the study population (N = 149).

Variable	N	%
**Age, mean ± SD**	61.1 ± 12.6	
**Sex**		
Male	107	71.8
Female	42	28.2
**Smoking status**		
Never smoker	61	40.9
Ever smoker	65	43.6
Not recorded	23	15.4
**Histological subtype**		
Clear cell	109	73.2
Papillary	14	9.4
Chromophobe	4	2.7
Collecting duct	1	0.7
Mixed	2	1.3
Other	19	12.7
**Sarcomatoid differentiation**	16	10.7
**ECOG performance status**		
0	7	4.7
1	117	78.5
2	25	16.8
**Metastatic sites**		
Soft tissue	102	68.5
Lung	89	59.7
Bone	67	45.0
Liver	28	18.8
Central nervous system	13	8.7
**Number of metastatic regions**		
1	52	34.9
2	49	32.9
3	38	25.5
4	10	6.7
**First-line TKI agent**		
Pazopanib	74	49.7
Sunitinib	53	35.6
Cabozantinib	22	14.8
**Modified 5-factor IMDC descriptive category**		
Favorable	53	35.6
Intermediate	81	54.4
Poor	15	10.1
**RMH score**		
0	53	35.6
1	57	38.3
2	32	21.5
3	7	4.6
**Prior nephrectomy**		
Yes	90	60.4
No	59	39.6
**Mortality**		
Alive	50	33.6
Dead	99	66.4

TKI, tyrosine kinase inhibitor; IMDC, International Metastatic RCC Database Consortium; RMH, Royal Marsden Hospital; SD, standard deviation. Ever smoker includes ex-smokers and current smokers. In this exclusively de novo metastatic cohort, the IMDC component “time from diagnosis to treatment initiation < 1 year” was positive in all patients and therefore had no discriminatory value. Modified five-factor IMDC descriptive categories were derived by excluding this universally positive component. These categories are presented for descriptive purposes only and are not equivalent to standard six-factor IMDC risk categories.

**Table 2:** Univariate and multivariate Cox proportional hazards regression analyses for overall survival.

	Univariate			Multivariate		
Variable	*p*-Value	HR	95% CI	*p*-Value	HR	95% CI
Age	0.431	1.01	0.99–1.02	—	—	—
Male sex	0.165	1.39	0.87–2.21	0.141	1.45	0.88–2.38
Non-clear cell histology	0.242	1.30	0.84–2.00	—	—	—
Sarcomatoid differentiation	0.078	1.69	0.93–3.07	0.025	2.01	1.09–3.72
IMDC score	0.090	1.17	0.97–1.41	0.129	1.16	0.95–1.42
RMH score	0.016	1.30	1.05–1.61	0.026	1.29	1.03–1.61
Prior nephrectomy	0.052	0.66	0.44–1.01	0.077	0.67	0.42–1.05

HR, hazard ratio; CI, confidence interval; IMDC, International Metastatic RCC Database Consortium; RMH, Royal Marsden Hospital. Covariates were selected on the basis of a priori clinical relevance and statistical parsimony.

**Table 3:** Univariate and multivariate Cox proportional hazards regression analyses for progression-free survival.

	Univariate			Multivariate		
Variable	*p*-Value	HR	95% CI	*p*-Value	HR	95% CI
Age	0.441	0.99	0.98–1.01	—	—	—
Male sex	0.040	1.54	1.02–2.34	0.057	1.53	0.99–2.38
Non-clear cell histology	0.599	0.89	0.59–1.34	—	—	—
Sarcomatoid differentiation	0.090	1.60	0.92–2.78	0.074	1.65	0.95–2.87
IMDC score	0.743	0.97	0.82–1.16	—	—	—
RMH score	0.052	1.22	0.99–1.49	0.056	1.23	0.99–1.52

## Data Availability

The data are available upon request from the corresponding author.

## Supplementary Materials

The following supporting information can be downloaded at https://www.mdpi.com/article/10.3390/jcm15103613/s1. Table S1: Frequency of individual IMDC prognostic score components in the study cohort (N = 149).
