# Supplementary material for: The Royal Marsden Hospital Score Independently Predicts Overall Survival in Patients with De Novo Metastatic Renal Cell Carcinoma Treated with First-Line Tyrosine Kinase Inhibitors: A Multicenter Retrospective Analysis
Source: J Clin Med. 2026 May 8;15(10):3613. doi: 10.3390/jcm15103613 (PMC13207480; doi:10.3390/jcm15103613)
Supplement: Supplementary file 1 [file jcm-15-03613-s001.zip › jcm-4243864-supplementary.pdf]

**Supplementary Table S1.** Frequency of individual IMDC prognostic score components in the study cohort (N = 149).

| IMDC Component | N | % |
|---|---|---|
| Time from diagnosis to treatment initiation < 1 year | 149 | 100.0 |
| ECOG performance status ≥2 (Karnofsky performance status < 80%) | 25 | 16.8 |
| Hemoglobin below the lower limit of normal | 69 | 46.3 |
| Corrected serum calcium above the upper limit of normal | 14 | 9.4 |
| Neutrophil count above the upper limit of normal | 23 | 15.4 |
| Platelet count above the upper limit of normal | 37 | 24.8 |

*IMDC, International Metastatic Renal Cell Carcinoma Database Consortium; ECOG, Eastern Cooperative Oncology Group. Because the study was restricted to patients with de novo metastatic disease, the time from diagnosis to treatment initiation was less than one year in all patients; this component therefore did not contribute discriminatory information within this cohort.* Accordingly, the modified 5-factor IMDC descriptive categories reported in Table 1 and Figure 3 of the main manuscript were derived from the remaining five IMDC components, excluding this universally positive variable. These categories are presented for descriptive purposes only and should not be interpreted as standard six-factor IMDC risk categories.
